# Identification and characterization of a minisatellite contained within a novel miniature inverted-repeat transposable element (MITE) of *Porphyromonas gingivalis*

**DOI:** 10.1186/s13100-015-0049-1

**Published:** 2015-10-06

**Authors:** Brian A. Klein, Tsute Chen, Jodie C. Scott, Andrea L. Koenigsberg, Margaret J. Duncan, Linden T. Hu

**Affiliations:** Department of Molecular Biology and Microbiology, Tufts University Sackler School of Biomedical Sciences, Boston, MA 02111 USA; Department of Microbiology, The Forsyth Institute, Cambridge, MA 02142 USA

**Keywords:** Species-specific repeat, DNA structure, Miniature Inverted-repeat Transposable Element, BrickBuilt, Transcriptional regulation, *Porphyromonas*

## Abstract

**Background:**

Repetitive regions of DNA and transposable elements have been found to constitute large percentages of eukaryotic and prokaryotic genomes. Such elements are known to be involved in transcriptional regulation, host-pathogen interactions and genome evolution.

**Results:**

We identified a minisatellite contained within a miniature inverted-repeat transposable element (MITE) in *Porphyromonas gingivalis*. The *P. gingivalis* minisatellite and associated MITE, named ‘BrickBuilt’, comprises a tandemly repeating twenty-three nucleotide DNA sequence lacking spacer regions between repeats, and with flanking ‘leader’ and ‘tail’ subunits that include small inverted-repeat ends. Forms of the BrickBuilt MITE are found 19 times in the genome of *P. gingivalis* strain ATCC 33277, and also multiple times within the strains W83, TDC60, HG66 and JCVI SC001. BrickBuilt is always located intergenically ranging between 49 and 591 nucleotides from the nearest upstream and downstream coding sequences. Segments of BrickBuilt contain promoter elements with bidirectional transcription capabilities.

**Conclusions:**

We performed a bioinformatic analysis of BrickBuilt utilizing existing whole genome sequencing, microarray and RNAseq data, as well as performing *in vitro* promoter probe assays to determine potential roles, mechanisms and regulation of the expression of these elements and their affect on surrounding loci. The multiplicity, localization and limited host range nature of MITEs and MITE-like elements in *P. gingivalis* suggest that these elements may play an important role in facilitating genome evolution as well as modulating the transcriptional regulatory system.

**Electronic supplementary material:**

The online version of this article (doi:10.1186/s13100-015-0049-1) contains supplementary material, which is available to authorized users.

## Background

*Porphyromonas gingivalis*, a gram-negative, anaerobic, asaccharolytic, black-pigmenting bacterium, is a keystone pathogen in the development and progression of periodontal disease [1, 2]. Multiple repetitive and transposable elements were previously identified in the *P. gingivalis* genomes [3–12]. Genome sequences are now available for multiple strains of *P. gingivalis* which has greatly facilitated genetic and genomic analyses of the species [9–16]. Each of the sequenced *P. gingivalis* genomes has contained multiple repetitive and transposable elements, an aspect that makes sequencing and alignment difficult.

Repetitive Elements (REs) are DNA sequences present in multiple copies throughout a genome, chromosome or vector. They are broadly classified into ‘terminal’, ‘tandem’ and ‘interspersed’ repeats, however, each of these classifications encompasses several sub-types of REs. Tandem repeats are classified as either identical or non-identical based on the level of nucleic acid matching. They are then further classified as either micro, mini or macro satellites based on size of the repeat. Repetitive elements can either be localized at a single site where a motif is recurrent sequentially adjacent to each other or at many loci as reiteration [17–19].

Transposable Elements (TEs) are ‘mobile’ DNA sequences that can change locus or multiply and insert into new loci within a genome or between genomes via excision/replication and insertion. They can insert into chromosomes, plasmids and bacteriophages. Class I TEs are retrotransposons, which require reverse-transcriptase activity to transpose. Class II TEs are DNA transposons, which unlike reverse transcriptase-utilizing Class I elements, require a transposase or a replicase to transpose [19–21]. Class II elements can either be autonomous or non-autonomous, the latter [canonically] having undergone mutations involving the transposase such that they can no longer duplicate or excise without the assistance of a parent element that utilizes a similar transposase. Within the non-autonomous element sub-class are miniature inverted-repeat transposable elements, or MITEs [22–25].

MITEs have a distinct structure relative to other TEs. They are between 50–1000 bp in length and are often present in high copy numbers per genome. MITEs are typically AT-nucleotide (nt) rich and frequently contain terminal inverted repeats (TIRs) and target site duplications (TSDs), but they lack the capacity to code for functional transposases [22–25]. Transposable elements, in particular MITEs, can be found in all taxa, varying in number and type between species and can account for greater than half of a genome. Bacteria typically carry between 10–20 copies of a MITE per genome, while plants may have up to 20,000 copies of a given MITE. Copy numbers are suggested to depend on non-coding region availability, polyploidy, the presence of a fully-functional autonomous version of a transposase, evolutionary ‘burst’ opportunities and regulatory potential of the given element [26–29]. Eukaryotic MITEs are frequently found in or closely associated with the coding region while prokaryotic MITEs are almost exclusively found intergenically [26, 30–36]. Intergenically located MITEs in prokaryotes have been shown to be able to affect gene expression [23, 25].

Several studies have demonstrated potential interactions of repetitive elements with transposable elements, which are generally thought to work independently and be mutually exclusive. In the wedge clam (*Donax trunculu*) genome as well as the butterfly and moth (*Lepidoptera*) genomes, ‘hitchhiking’ microsatellites were found within transposable elements [37, 38]. Microsatellites and simple sequence repeats have also been found closely associated with transposable elements in *Neisseria meningitidis* [39].

Here we describe ‘BrickBuilt’, a miniature inverted-repeat transposable element containing a minisatellite, in *P. gingivalis*. The sequences, location, copy number, prevalence throughout the species, as well as implications on genome (in)stability and transcriptional regulation are described. Similarities to other autonomous and non-autonomous *P. gingivalis* transposable elements are addressed with the goal of defining a potential network for the biogenesis of these elements in *P. gingivalis* and their effects on the *P. gingivalis* genome.

## Results and discussion

### Identification of a repetitive element in *Porphyromonas gingivalis*

**Fig. 1:**
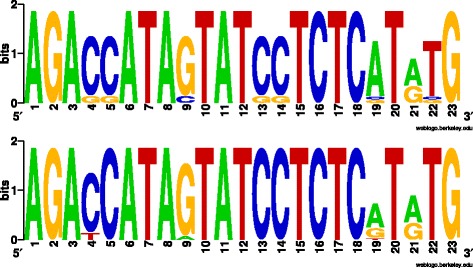
Sequence logo representation of the 23 nucleotide repeat region from *P. gingivalis* strain ATCC 33277 multiple alignments. Generated with Weblogo software. Total height of a nucleic acid stack represents sequence conservation at a given position. Height of symbols at a stack represents relative frequency of a given nucleic acid at that position. Top sequence logo corresponds to compilation of the consensus of the 19 BrickBuilt elements; consensus for each made prior to combining for sequence logo. Bottom sequence logo corresponds to BrickBuilt_5 alone, constructed from its 18 full repeats

We identified a DNA element, ‘BrickBuilt’, in the genome of *P. gingivalis* strain ATCC 33277. The element was initially identified as a tandemly-repeated sequence of 23 nt located intergenically at a single site (Additional file 1: Figure S1). A more thorough investigation of the genome revealed 19 independent, non-identical segments of the element scattered throughout the genome of strain ATCC 33277 (Table 1). The smallest number of 23 nt direct repeats is 1 (BrickBuilt_1) and the largest 22.8 (BrickBuilt_12). The 23 nt direct repeats are imperfect within a given element, imperfect bases vary from one element to another and imperfections do not correlate with length or total number of repeats within a given element (Fig. 1). The percent of mismatches within a given element varies from 0 to 11, and the percent of insertions and deletions within an element varies from 0 to 6. Within the 23 nt repeats there are conserved and non-conserved nucleotide sites, with the latter half of the element containing the majority of non-conserved sites (Fig. 1). Although similar in length to CRISPR element spacers and microRNAs, BrickBuilt elements are seemingly unrelated to these other entities.

**Table 1:** Genes/Coding Sequences located 5′ and 3′ to BrickBuilt elements. Gene numbers and characterizations correspond to strain ATCC 33277. Loci of BrickBuilt elements across four sequenced and annotated *P. gingivalis* strains. BrickBuilt elements are situated intergenically between the genes noted. Grayed-out boxes represent loci at which BrickBuilt is aberrant

MITE	33277 Locus	W83 Locus	TDC60 Locus	HG66 Locus	Gene Characterization	Gene	Strand
BrickBuilt_1	PGN_0031	PG0033	PGTDC60_0032	EG14_02395	RmuC domain (DUF 805)		-
	PGN_0033	PG0034	PGTDC60_0034	EG14_02400	Thioredoxin	*trx*	-
BrickBuilt_2	PGN_0204	PG2159	PGTDC60_1262	EG14_03005	Protoporphyrinogen oxidase	*hemG*	+
	PGN_0205	PG2161	PGTDC60_1265	EG14_03010	AraC family transcriptional regulator		-
BrickBuilt_3	PGN_0303	PG0196	PGTDC60_0466	EG14_04795	Zinc protease (Peptidase M16)		+
	PGN_0306	PG0198	PGTDC60_0471	EG14_04805	PF05656 family protein (DUF 805)		+
BrickBuilt_4	PGN_0336	**NP**	PGTDC60_1661	EG14_04940	Immunoreactive antigen/PorSS CTD		-
	PGN_0340	**NP**	PGTDC60_1665	EG14_04960	Peptidase S41/PorSS CTD		+
BrickBuilt_5	PGN_0361	PG0264	PGTDC60_0543	EG14_05065	Glycosyl transferase family 2		+
	PGN_0365	PG0267	PGTDC60_0547	EG14_05075	Arginyl-tRNA synthetase	*argS*	+
BrickBuilt_6	PGN_0400	PG1715	PGTDC60_0586	EG14_05255	TonB-dependent receptor Cna protein		+
	PGN_0403	PG1714	PGTDC60_0590	EG14_05265	Pyridoxamine-phosphate oxidase	*pdxH*	+
BrickBuilt_7	PGN_0455	PG0549	PGTDC60_0639	EG14_03135	Partial ISPg5		+
	PGN_0456	PG0553	PGTDC60_0641	EG14_03150	Methylmalonyl-CoA mutase	*scpA*	-
BrickBuilt_8	PGN_0550	PG1559	PGTDC60_0739	EG14_03610	Glycine cleavage system subunit T	*gcvT*	+
	PGN_0553	PG1556	PGTDC60_0743	EG14_03615	Conserved hypothetical (DUF2149)		-
BrickBuilt_9	PGN_0558	PG1548	PGTDC60_0748	EG14_03640	Haem-binding protein	*hmuY*	-
	PGN_0559	PG1550	PGTDC60_0751	EG14_03655	Serine protease (Peptidase C10)	*prtT*	-
BrickBuilt_10	PGN_0632	PG0585	PGTDC60_1709	EG14_09225	Aspartyl-tRNA amidotransferase B		+
	PGN_0633	PG0587	PGTDC60_1713	EG14_09235	Membrane protein putative ion channel	*btuF*	-
BrickBuilt_11	PGN_0667	PG0625	PGTDC60_1753	EG14_09060	GTP cyclohydrolase I/PorSS CTD	*folE*	+
	PGN_0668	PG0627	PGTDC60_1756	EG14_09045	RNA-binding protein/PorSS CTD		-
BrickBuilt_12	PGN_0819	PG0796	PGTDC60_1912	EG14_08255	Leucyl-tRNA synthetase	*leuS*	-
	PGN_0823	PG0800	PGTDC60_1917	EG14_08240	NAD-utilizing dehydrogenase		-
BrickBuilt_13	PGN_0831	PG0807	PGTDC60_1926	EG14_08205	N utilization substance/PorSS CTD		-
	PGN_0832	PG0809	PGTDC60_1927	EG14_08200	Gliding motility protein/PorSS CTD	*sprA*	-
BrickBuilt_14	PGN_0871	PG1389	PGTDC60_2074	EG14_07995	Membrane protein		-
	PGN_0872	PG1391	PGTDC60_2073	EG14_08000	DNA-binding protein (PF00216)		+
BrickBuilt_15	PGN_0898	PG1424	PGTDC60_2039	EG14_07870	Peptidylarginine deiminase/PorSS CTD	*PAD*	-
	PGN_0900	PG1427	PGTDC60_2036	EG14_07865	Peptidase C10/PorSS CTD		-
BrickBuilt_16	PGN_1207	PG1117	PGTDC60_1098	EG14_06320	Transport multidrug efflux		+
	PGN_1208	PG1118	PGTDC60_1096	EG14_06310	ClpB chaperone and protease	*clpB*	-
BrickBuilt_17	PGN_1476	PG0494	PGTDC60_1611	EG14_09640	PorSS C-terminal sorting domain		-
	PGN_1479	PG0491	PGTDC60_1606	EG14_09650	Peptidase S10/PorSS CTD	*dppVII*	-
BrickBuilt_18	PGN_1777	PG1784	PGTDC60_0106	EG14_00235	Cysteine protease (Peptidase C1)		-
	PGN_1780	PG1786	PGTDC60_0110	EG14_00245	Endoribonuclease L-PSP		-
BrickBuilt_19	PGN_2035	PG0088	PGTDC60_0367	EG14_01925	Peptidase M16		+
	PGN_2037	PG0090	PGTDC60_0370	EG14_01930	DNA-binding protein from starved cells	*dps*	+

‘*NP*’ stands for ‘Not Present’

**Fig. 2:**
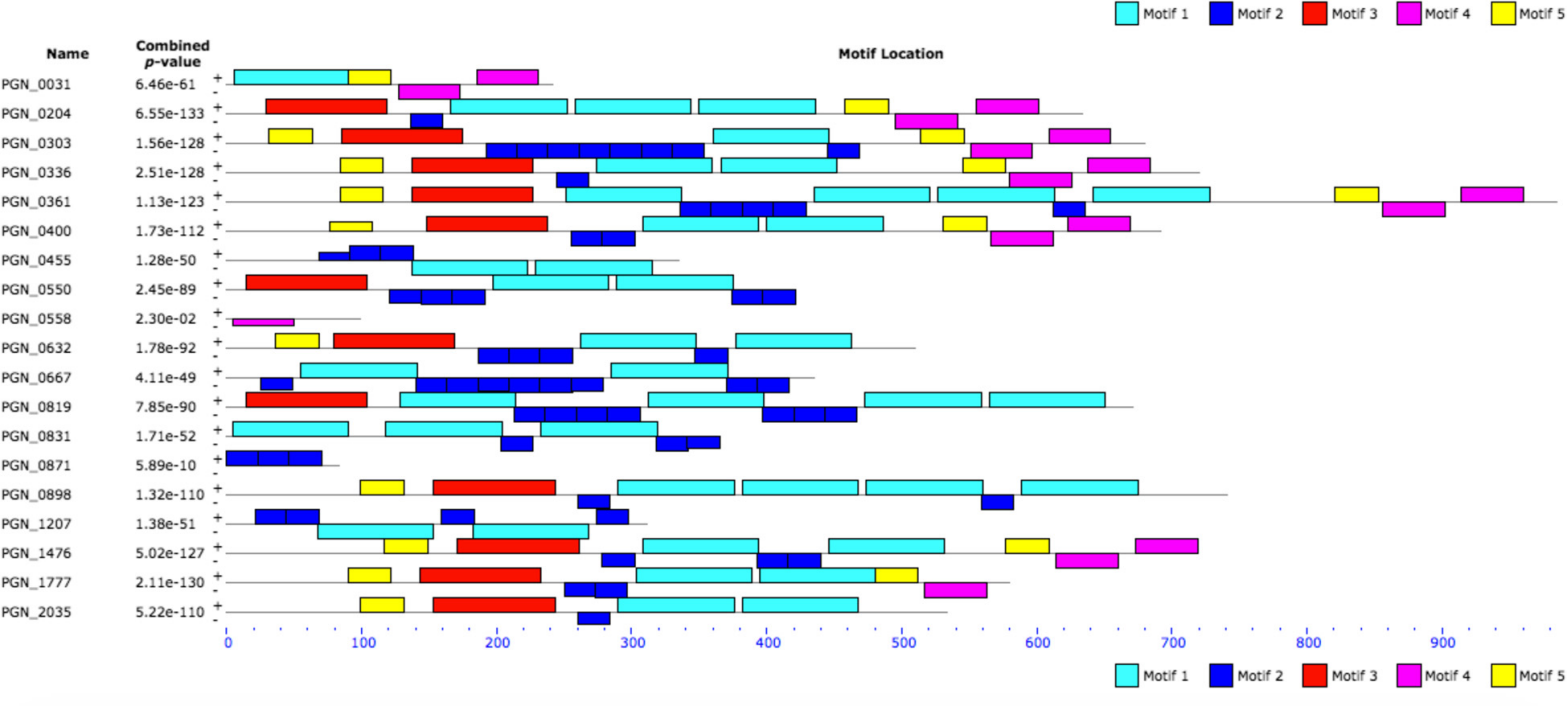
MEME motif analysis block output of the 19 BrickBuilt elements in *P. gingivalis* strain ATCC 33277. Entire element FASTA sequences were used as the input. Settings for analysis were: Distribution of motif occurrence as ‘any number of repetitions’, number of different motifs as ‘5’, minimum motif with as ‘23’, and maximum motif width as ‘200’. Dark and light blue blocks correspond to 23 nucleotide repeat regions, red blocks to leader regions and purple blocks to tail regions. Yellow blocks, potentially representing a 5th motif, were only found on the positive strand and had the lowest e-values associated with their significance scores

After determining the length and locations of each independent direct repeat element we performed alignments of the sequences flanking the direct repeats to determine whether specific DNA sequences or motifs were necessary for the presence of the element. Alignments of the sequences flanking the direct repeats revealed regions of homology, different for the two flanks of the repeat, which were determined to be ‘leader’ and ‘tail’ regions that encompassed the direct repeats (Fig. 2). Of the 19 elements, 11 are flanked by portions of both a leader and a tail, 3 by just leader, 2 by just tail, and 3 by neither. When considered as a single whole element, all BrickBuilt elements are intergenic, although some are within regions where annotation pipelines predicted hypothetical genes that do not appear to be expressed based on proteomic data [40–42]. Total length of the complete elements ranges from 991 nt (BrickBuilt_5) to 84 nt (BrickBuilt_14), which is determined by number of internal 23 nt repeats as well as the specific element may contain full, partial or no leader and tail segments. The longest leader segment is 285 nucleotides (BrickBuilt_17) and the longest tail segment is 318 nucleotides (BrickBuilt_4). No BrickBuilt element is bisected by a full-length autonomous transposable element. Thus, although repetitive intergenic sequences may be targets for insertion of exogenous or duplicated endogenous genes, such events have yet to be detected. Additionally, no leader-to-tail versions are present without a full 23 nt repeat and no TIR-containing individual leader or tail segments are present without the 23 nt repeats. A single site adjacent to the Hmu operon contains a partial tail-only version that lacks the terminal 20 nt that would include the TIR; no partial leader-only versions of the element are present.

The genome sequence of strain ATCC 33277 contains 2,345,886 bases. When complied together all 19 BrickBuilt elements in strain ATCC 33277 make up 10,276 bases, or 0.44 percent of the overall genome; the equivalent of 9 protein coding sequences in this strain on average.

### Conservation of BrickBuilt elements in other strains of *P. gingivalis*

Of the 19 versions of BrickBuilt found within strain ATCC 33277, 16 are conserved between the analogous coding sequences within strains W83, TDC60 and HG66 (Table 1 and Additional file 2: Table S1). Strains HG66 and TDC60 contain 19 versions of BrickBuilt, equivalent to the number in strain ATCC 33277. However, strain W83 only contains 18 versions of the element. Strains ATCC 33277 and HG66 share the exact same 19 loci for BrickBuilt elements. One locus in strain TDC60 (BrickBuilt_4) is deviant and is encompassed by two IS*Pg*1 elements. Strain W83 has three sites that differ from the other strains, all which are located adjacent to other types of IS or repetitive elements. In this strain BrickBuilt_4 is completely lacking, while BrickBuilt_7 and BrickBuilt_18 are aberrant with respect to size having only maintained 23 nt repeats.

BrickBuilt elements can be identified in the genome of *P. gingivalis* strain JCVI SC001, which is not yet included in the default NCBI BLAST nucleotide database settings. Genome searches of the FASTA files from the JCVI SC001 revealed that most BrickBuilt loci in the JCVI SC001 genome contained strings of undetermined bases, which can be attributed to the manner of isolation, sequencing and assembly. Eight other *P. gingivalis* genomes have since been sequenced and deposited in NCBI, yet they are not completely assembled. Assembly gaps are located at sites where the corresponding surrounding CDS from ATCC 33277 contain BrickBuilt elements, suggesting BrickBuilt is present in those genomes as well and potentially capable of causing assembly difficulties (Additional file 3: Figure S2). Additionally, a degenerate version of the 23 nt repeat consensus sequence (AGAYCATARTATCCTCTCRTRTG) was searched against all 13 (8 unfinished) *P. gingivalis* genomes, each giving positive hits (data not shown). Because of assembly gaps and undetermined bases around BrickBuilt sites in the unfinished sequencing projects they were not included in multiple alignments.

**Fig. 3:**
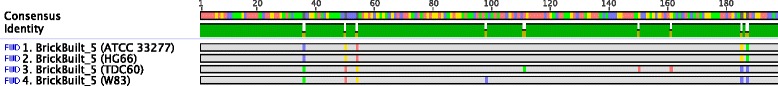
BrickBuilt_5 multiple alignment generated using MAFFT on the Geneious R8 platform with strain ATCC 33277, W83, TDC60 and HG66 inputs. The multiple alignment is focused on the first 200 nt of the leader region. Grey within the tracks denotes complete conservation at a site. Color within the tracks denotes variable sites. At all five sites where more than one strain is variable strains ATCC 33277 and HG66 cluster together, as do strains TDC60 and W83. Within this region only strains TDC60 and W83 have sites where they alone differ from the other three strains; this is consistent throughout for this specific element

Multiple alignments of BrickBuilt elements using the sequences from strains ATCC 33277, W83, TDC60 and HG66 revealed that sequences from strain ATCC 33277 align most closely with HG66, and those from strain W83 with TDC60 (Fig. 3). Similarly, a phylogenetic analysis with PHYML showed similar clustering of ATCC33277 with HG66 and W83 with TDC60 (Fig. 3). The matching of the sequences between the strains in the above pairings is consistent throughout 18 of 19 elements. Branching of BrickBuilt elements is congruent with the dendrogram generated based on genomic BLAST for all 13 *P. gingivalis* genomes. Interestingly, strain HG66 was deposited as ‘being closely related to strain W83’, yet based on the results of dendrogram from the full genome BLAST and from alignments of the BrickBuilt, this seems to be incorrect. The BrickBuilt_5, BrickBuilt_8, BrickBuilt_10, BrickBuilt_11, BrickBuilt_12, BrickBuilt_13, BrickBuilt_14 and BrickBuilt_15 sites all lie between the same two CDS within the respective genomes, with strains ATCC 33277 and HG66 usually having more 23 nucleotide repeats than W83 and TDC60. BrickBuilt_6 is the only site at which strains W83 and TDC60 have more 23 nt repeats than ATCC 33277 and HG66. BrickBuilt_9, the shortest element which is also the only element without a 23 nt repeat has only one SNP across the 100 nucleotides. Unlike all the other BrickBuilts, that single SNP would align strain ATCC 33277 with W83 and strain TDC60 with HG66.

The repetitive nature of the BrickBuilt elements, both the internal repeats and that they are found multiple times through the genome, can lead to sequencing, assembly and annotation issues. Because the strains W83, ATCC 33277, TDC60, HG66 and JCVI SC001 are unique strains, were sequenced independently, and were *de novo* assembled, placement of BrickBuilt elements at the same locus across genomes is unlikely to be due to use of a shared scaffold. However, care should be taken when aligning newly-sequenced *P. gingivalis* genomes to a scaffold.

### Homology to MITEs and other repetitive elements

The 23 nt repeats and the leader and tail segments of the element were analyzed using BLAST (NCBI server) to determine whether the element is present in genomes other than the species *P. gingivalis* [43]. With default BLAST nucleotide settings, a full-length BrickBuilt and each of the three distinct parts of the element match solely to *P. gingivalis*. All four sequenced and annotated strains of *P. gingivalis* available for BLAST searching harbored hits for BrickBuilt. Through querying discontiguous megablast as well as using less stringent search constraints within megablast with ‘max target sequences’, ‘expect threshold’, ‘word size’ and ‘filter low complexity regions’, low-homology hits were obtained with the terminal inverted repeat regions. However, matches identified in this manner were only homologous in the TIR sections. Of note, when BLASTx searches (protein database search using a translated nucleotide query) were performed with the leader and tail sequences under default settings several *Bacteroidetes* species contained tail hits and one species contained a leader hit. *Porphyromonas gulae* contained strong hits with both leader and tail, while *Prevotella tannerae* and *P. dentalis* contained weak tail hits only. All of the BLASTx hits were either part of a predicted transposase/partial transposase or a hypothetical protein. If BrickBuilt were indeed a non-autonomous transposable element, homology to sections of related transposons through BLASTx would not be unexpected. As such, low BLASTx homology within *Prevotella tannerae* and *P. dentalis* does not point to these hits being potential parent or identical elements of BrickBuilt.

Genome analysis of recently-uploaded *Porphyromonas gulae* strains was carried out using the NCBI-deposited WGS shotgun sequencing data, from which we determined that *P. gulae* strains do in fact carry BrickBuilt homologues (Additional file 4: Figure S3) [44, 45]. Some of the *P. gingivalis* BrickBuilt element locations are conserved within *P. gulae* strains. However, greater strain variation seems evident in *P. gulae* at certain BrickBuilt loci than between *P. gingivalis* strains (Additioal file 5: Figure S3). Of note, the original *P. gulae* genome was obtained from a wolf and the subsequent strains were obtained from domesticated dogs. The original strain (DSM 15663) only contains 4 BrickBuilt homologues within the genome, and importantly lacks the BrickBuilt_5 homologue that was used for the majority of BLAST database queries.

Within *P. gingivalis* there have been three previously identified groups of MITEs or non-autonomous transposable elements; named the 239, 464 (PgRS) and 700 groups [10, 13]. These numbers are the names of three types of MITEs already noted in *P. gingivalis* genomics publications. The numbers were initially related to the overall length of the elements, however, the 464 type was renamed in subsequent publications and in NCBI genome graphics annotations. General copy numbers of the four MITE versions are similar, holding around 10–20. The number of full copies and partial or fragment copies of each element differs slightly between genomes within the species. During our examination of BrickBuilt we analyzed whether any sequence overlap was apparent between the elements and found that the terminal inverted repeats are similar, yet the rest of the elements do not bear similarity. 464/PgRS elements were previously identified as containing 41 nucleotide tandem direct repeats [10, 13]. The 23 and 41 nucleotide internal tandem direct repeats of the elements do not share homology with each other and neither have non-*P. gingivalis* BLAST matches within the NCBI database. The segments of the 464/PgRS elements flanking the 41 nt tandem direct repeats are themselves repetitive, which is unlike the non-repetitive leader and tail segments of BrickBuilt. Although not related by sequence, similarities in copy number between 464/PgRS and BrickBuilt elements are evident. With *P. gingivalis* harboring four types of MITE-like elements it is interesting that two types, BrickBuilt and 464/PgRS, contain microsatellite repeats. Although several 236 and 700-type elements are located near repeats or other repetitive elements, they seem not to have encompassed any mini- or microsatellites from analyses of the currently available genomes.

In addition to Tn and IS elements, multiple groups have described repetitive sequences within *P. gingivalis* genomes ranging from single nucleotide tracts to mini- and microsatellites [13, 46]. Several 41, 23 and 22 nucleotide tandem direct repeats were described in *P. gingivalis* strain W83, yet the exact locations of such repeats were not identified, nor were comparative genomics an option at the time of the report [13]. Some of the 23 nt tandem direct repeats noted are presumably the direct repeat portions of BrickBuilt.

**Fig. 4:**
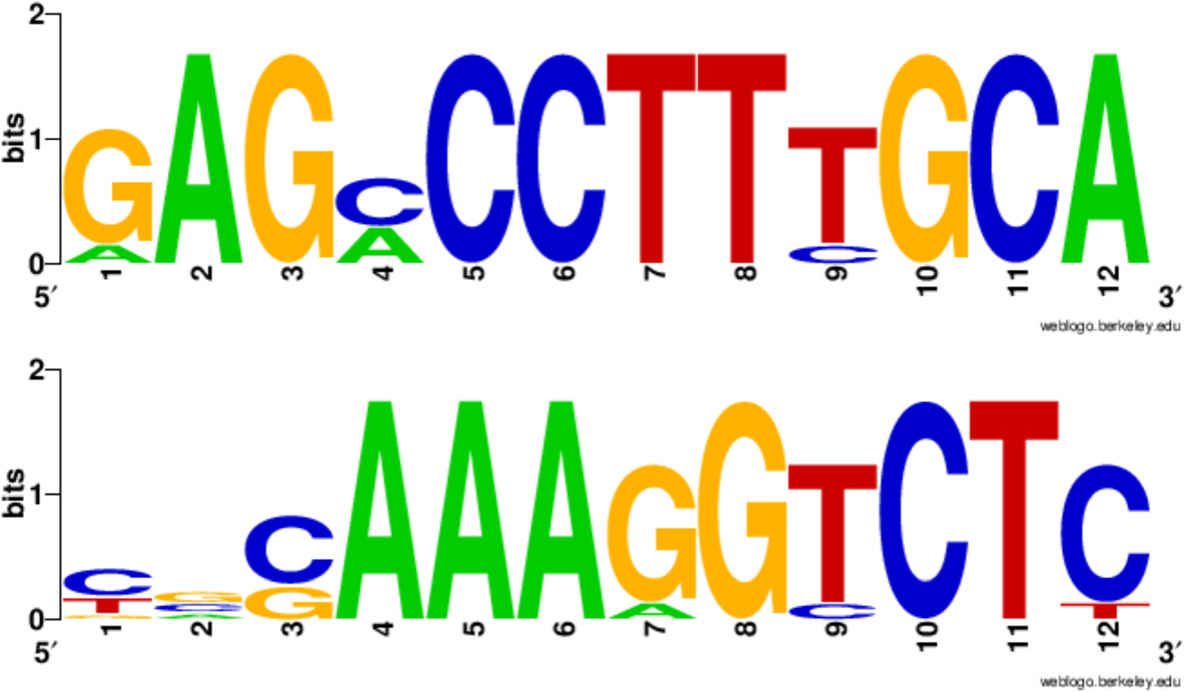
Consensus Terminal Inverted Repeats (TIRs) of BrickBuilt elements visualized with Weblogo software. Only elements that contained intact leader or tail segments, which carry the TIRs, were considered and used for consensus construction

Within *P. gingivalis* there are 11 recognized IS elements and 2 different composite transposon (Ctn) elements [13, 47, 48]. Ten of the terminal inverted repeats for the 13 IS and Ctn have been previously characterized. The TIRs of BrickBuilt were identified by first determining where non-repetitive DNA sequences immediately flanked repetitive ones (i.e. leader and tail segments). Next, all sequences were compared in multiple alignments, and only versions that maintained intact leaders or tails were then used for determination of consensus sequences (Fig. 4). The TIRs of BrickBuilt and MITEPgRS elements are almost identical, as are the TIRs of MITE293 and MITE700 elements. The MITE-like elements in *P. gingivalis* share either identical or within one nucleotide TIRs with those of full-length IS elements within the *P. gingivalis* genomes; ISPg1, ISPg3, ISPg4 and ISPg9 (Table 2). The matching full-length ISPg elements are all categorized within the IS5 family. BrickBuilt’s TIRs are most similar to those of ISPg1 and ISPg9 (which share identical TIRs); ISPg4 is the next closest match with 2 nucleotides different (Table 2). MITE293 and MITE700 TIRs match with ISPg3. Although the TIRs are similar, no remnant of a transposase from any *P. gingivalis* IS or Tn element remains within any of the BrickBuilt copies.

**Table 2:** Terminal Inverted Repeats (TIRs) of BrickBuilt elements from strain ATCC 33277. Terminal Inverted Repeats and family of selected IS and MITE-like elements in *Porphyromonas gingivalis* as well as ISNme1 from *Neisseria meningitidis*

Locus (33277)	TIR 5′ (nt)	TIR 3′ (nt)	Both TIRs
BrickBuilt_1		CCGAAAGGTCTC	
BrickBuilt_2		CCGAAAGGTCTC	
BrickBuilt_3	AAGACCTTTGCA	CCGAAAGGTCTC	YES
BrickBuilt_4	GAGACCTTTGCA	TGCAAAGGTCTC	YES
BrickBuilt_5	GAGACCTTTGCA	CGCAAAGGTCTC	YES
BrickBuilt_6		CACAAAGGTCTT	
BrickBuilt_7			
BrickBuilt_8		TGCAAAAGTCTC	
BrickBuilt_9			
BrickBuilt_10			
BrickBuilt_11			
BrickBuilt_12		GACAAAGGTCTC	
BrickBuilt_13			
BrickBuilt_14			
BrickBuilt_15	GAGCCCTTTGCA		
BrickBuilt_16			
BrickBuilt_17	GAGCCCTTCGCA		
BrickBuilt_18	GAGCCCTTTGCA	TGCAAAGGCCTC	YES
BrickBuilt_19	GAGCCCTTTGCA		
Element	Left TIR	Right TIR	Family
ISPg1	GAGACCATTGCA	TTCAAAGGTCTC	IS5
ISPg3	ACGTCAGTTCGA	TCGAACTGACGT	IS5
ISPg4	GAGACTGTTGCA	CGCAACAGTCTC	IS5
ISPg9	GAGACCATTGCA		IS5
MITE239	ACGTGAGTTCGATATAAAGGAA	TTCGCTTAAATCGAACTGGCGT	
MITEPgRS/MITE464	GAGACTGTTGCA	TGCAACGGTCTC	
MITE700	ACGTCATTCGA	TCGAACTCACGT	
BrickBuilt	GAGACCTTCGCA	TGAAAGGTCTC	
Nsm ISNme1	GAGACCTTTGCAAAA	TTTTGCAAAGGTCTC	IS5

Blank spaces for TIRs indicate situations in which the region is degenerate or not present

Only 4 of the 19 BrickBuilt copies contain both an intact leader and tail associated TIR (Table 2). In order to determine whether BrickBuilt makes target site duplications (TSD), the DNA sequence immediately adjacent to the proposed TIRs were examined of these 4 copies. Three of the four copies of BrickBuilt do carry TSDs, however, they are not the same length or sequence for each element. BrickBuilt_3 has a ‘CT’ dinucleotide flanking its TIRs; BrickBuilt_4 has a ‘GAAA’ tetranucleotide flanking its TIRs; BrickBuilt_5 has an ‘AAAAA’ heptanucelotide; and BrickBuilt_18 does not contain a putative TSD because one of its two TIRs is shared by a MITE293 element. These duplications on either side of the elements may not reflect canonical TSDs, however, if these elements are mobilized by multiple transposases that each make different restrictions to the target DNA this could potentially occur. Within *P. gingvialis*, ISPg elements generate TSDs varying from 2–9 bp; some can lack TSDs completely and may frequently nest into other mobile elements and thus eliminate TSD identification. Additional TSD data related to element mobility will be presented below.

After determining the IS5 family-like TIRs of BrickBuilt other IS5 elements were scanned for potential similarity. Identical TIRs to that of BrickBuilt were found in the *Neisseria meningitidis* ISNmeI and its derivatives (Table 2). ISNmeI is the proposed (based on TIRs) parent element for the type II MITE ATR (*AT*-rich *R*epeat) in *Neisseria meningitidis* genomes [39]. ATR elements are found 19 times within *N. meningitidis* genomes, which is similar to that of BrickBuilt’s distribution. Additionally, ATR elements are frequently associated with direct repeat elements of *N. meningitidis* known as REP2.

From initial characterizations of the configuration and locations of BrickBuilt elements, they can be classified within the large group of non-autonomous transposable elements, potentially best fitting within the MITE subcategory. A caveat must be placed, however, given that MITE elements are typically described as being comprised of two homologous flanking regions, and we have determined that BrickBuilt elements contain distinct ‘leader’ and ‘tail’ segments. Since all accessible genome sequences of *P. gingivalis* strains contain BrickBuilt elements, the parent element or first version of BrickBuilt probably occurred early within the phylogeny of the *P. gingivalis* species. Insertion of the 23 nt repeat(s) into the original parent element may be the event that catalyzed the inactivation of an autonomous transposable element. Alternatively, a version of BrickBuilt already containing the 23 nt repeats could have been laterally-transferred via plasmid or horizontally-transferred via phage. Given that no full-length (TIR-containing) leader or tail regions are present without a 23 nt repeat it may be deleterious to maintain a full leader or tail region on the chromosome, or the 23 nt repeat is required by the autonomous element.

The limited host range nature of BrickBuilt identified through NCBI BLAST is intriguing yet not uncommon for non-autonomous transposable elements [49–52]. Once a non-autonomous element occurs within a genome, potentially by deletions of an autonomous transposable element, as well as via conjugation-based horizontal transfer of a plasmid or transduction via a bacteriophage, movement between species will become less likely. Additionally, few bacterial species have multiple genome assemblies available for intraspecies comparisons, which could lead to missed elements due to strain variation. Furthermore, it is possible that repetitive sequences could be mis-sequenced or left out of genome assemblies due to repeat region sequencing difficulties or unassigned bases.

### Predicted secondary structure of BrickBuilt

**Fig. 5:**
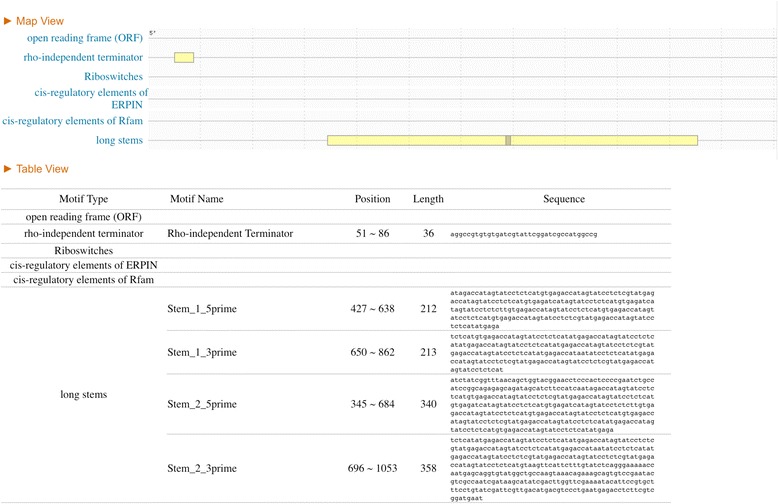
RegRNA2.0 analysis output of *P. gingivalis* strain ATCC 33277 BrickBuilt_5 and surrounding CDS-to-CDS area. The immediate 5′ CDS is PGN_0361 and the immediate 3′ CDS is PGN_0364 (*argS*). Options of open reading frames, Rho-independent terminators, transcriptional regulatory motifs, riboswitches, cis-regulatory elements, ERPINs, Rfam database matches, long stems and functional RNA sequences were queried. The Rho-independent terminate identified occurs prior to the BrickBuilt element

**Fig. 6:**
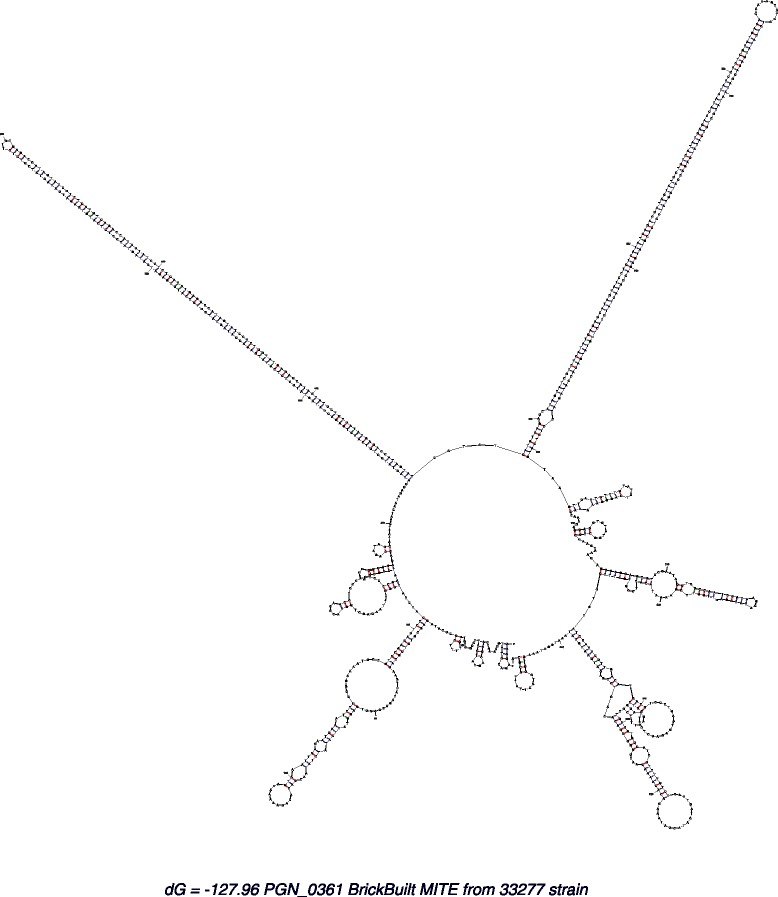
Mfold analysis output of BrickBuilt_5 for *P. gingivalis* strain ATCC 33277 (top) and W83 (bottom). Calculated entropy for the ATCC 33277 structure is ΔG = −128.02 and for W83 is ΔG = −101.06. Input for strain ATCC 33277 was 991 nt and 807 nt for strain W83. The 184 nt length difference is due to 23 nt repeat numbers. The 23 nt repeats comprise the long stem loops

The direct repeats within BrickBuilt are predicted to form long stem loop structures (Figs. 5 and 6). Three DNA/RNA structure prediction programs, Mfold, RNAstructure and RegRNA2.0, independently predicted long stem loops to form from/within the element [53–55]. The length of the version of BrickBuilt affects the size of the predicted stem loop structure and the associated entropy. BrickBuilt_1, BrickBuilt_9 and BrickBuilt_14 are not predicted to form long stem loops by the RegRNA2.0 program due to the length of the internal 23 nt repeats, however, shorter stem loops due to dyad symmetry may occur. BrickBuilt elements are predicted to be surrounded/flanked by Rho-independent terminators and/or polyadenyltaion sites in 10 of 19 instances. No portion of BrickBuilt matched to any structures in Rfam [56].

Predicted structures of BrickBuilt vary slightly between strains at a given conserved locus. The BrickBuilt_5 Mfold entropy predictions for strains ATCC 33277 and W83 are −127.96 and −102.50, respectively (Fig. 6). BrickBuilt_5 in ATCC 33277 is 991 nucleotides long and the analogous W83 version is 807 nucleotides. In this case the length difference is due to W83 BrickBuilt_5 having fewer 23 nt internal direct repeats; the leader and tails are of the same length. Within a multiple alignment of the four *P. gingivalis* strains at the BrickBuilt_5 locus there are 18 single nucleotide polymorphisms (SNPs) that separate the lineages (Fig. 6). Substituting SNPs between strains at the BrickBuilt_5 locus into the ATCC 33277 model changes the predicted entropy of the element by −7.25, or 5.7 %, to −135.17.

Although no BLASTn matches for BrickBuilt were found outside of the *P. gingivalis* species, MITEs from other species have been shown or are predicted to be of similar modular makeup and form long stem loops [23, 35, 38, 57–59]. In addition, repetitive sequences that are not MITE-associated also frequently form stem loop structures [58, 60–62]. Stem loop structures, especially long stem loops, are capable of modulating transcript half-life, modulating translational efficiency as well as serving as docking/receptor sites for proteins [12, 58, 61]. The Rho-independent terminator upstream of BrickBuilt_5 is located 111–148 nt from the 5′ CDS, with the leader region of BrickBuilt_5 located 182 nt from the 5′ CDS (Fig. 6). Thus, in this case, the BrickBuilt element has not disrupted the ‘natural’ terminator for the 5′ CDS. However, given the proximity to the Rho-independent terminator, this BrickBuilt element may be able to modulate the stability or accessibility of the terminator. The long stem loop structures of BrickBuilt_5 start 257 nt from the Rho-independent terminator and end 965 nt away.

### Genome locations and surroundings

All BrickBuilt elements are located intergenically; no direct overlap or interruptions of genuine protein coding sequences are apparent in the complete genomes available to date (Table 1 and Additional file 2: Table S1). Several ‘hypothetical proteins’ are annotated to be within BrickBuilt elements, however expression of these proteins has not been confirmed experimentally [40–42]. Several of the predicted hypothetical proteins are part of repeated/overlapping probes on *P. gingivalis* microarrays. Additionally, the 23 nt repeats within BrickBuilt elements are predicted to cause frequent translational stops (data not shown). Lack of experimental confirmation of protein products, nonunique microarray probes and abundant translational stops suggests that translation of these regions is unlikely, and even if translation were to occur it would probably be truncated versions of a repetitive or mobile element.

Of the 38 genes surrounding the BrickBuilt elements in the ATCC 33277 genome there are several functional clusters (Table 1). Six genes encode proteases of the C1, (2) C10, (2) M16 and S41 families. Five genes are predicted to encode DNA/RNA-binding proteins, and another four are involved in tRNA metabolism. Noticeably, of the 19 BrickBuilt elements in strain ATCC 33277, 5 are located adjacent to a gene/protein containing a Por Secretion System C-Terminal Domain (PorSS CTD) [63] (Table 1). Likewise, 5 of the previously identified *P. gingivalis* MITEs within the W83 genome are also located next to PorSS CTDs. A total of 34 PorSS CTDs have been predicted within the W83 genome (only 22 annotated on NCBI with TIGR); 29 % of PorSS CTDs are associated with MITEs [64]. The PorSS is connected to pigmentation and haem acquisition in *P. gingivalis*. Apart from those associated with PorSS, other genes surrounding BrickBuilt elements are *hemG*, *dps, trx, and hmuY,* which are involved in haem biosynthesis, acquisition and detoxification, respectively. Additionally, two separate DUF 805 motifs are found in genes surrounding BrickBuilt elements, which are associated with phage integrases. The locations relative to CDS raise the possibility that BrickBuilt could be acting as or similar to a Putative Mobile Promoter (PMP); a secondary regulatory circuit or mechanism to canonical transcription and translation modulation [65].

Expansion and contraction of the 23 nt repeats between strains is evident at the conserved BrickBuilt loci. Entire 23 nt repeat segments have been removed or added. Full and/or partial deletions of the leader and tail regions are also apparent. Deletions of the leader and tail regions occur from the distal ends of each segment with respect to the 23 nt internal repeats.

Pairwise and multiple alignments of a respective BrickBuilt locus across the four strains of *P. gingivalis* revealed SNPs that potentially suggest lineages or selected and compensatory mutations (Fig. 3). Whether the SNPs are generated *de novo* at each site, occur in stages and are distributed, or occur through site-to-site recombination cannot be determined definitively from currently published genome assemblies alone. However, multiple alignments of the conserved BrickBuilt loci within a given strain show patterns of non-random mutation. For sites at which SNPs have occurred, the SNP is frequently distributed at several positions within the element, yet this occurs at intervals. Additionally, SNPs appear localized around a 2–4 nt site when compared in multiple alignments. Long Term Evolution Experiments (LTEE) and plasmid-based recombination systems could be employed to determine mutation rates within BrickBuilt in comparison to the rest of the genome, whether 23 nt repeats expand and contract at a given locus, and how recombinogenic the elements are.

**Fig. 7:**
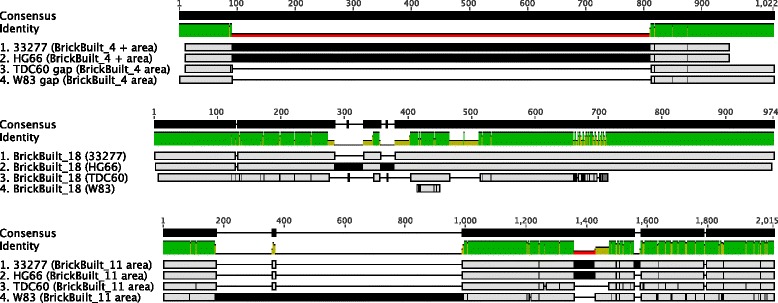
MAFFT-based alignments of aberrant BrickBuilt elements and areas across *P. gingivalis* strains. The top panel depicts the CDS-to-CDS region of BrickBuilt_4, using the surrounding CDS that would correctly correspond to the ATCC 33277 genome. Strain W83 has no BrickBuilt_4 and strain TDC60 has a BrickBuilt_4 that has moved or been moved to a different locus. The middle panel depicts BrickBuilt_18, in which strains ATCC 33277 and HG66 have a 236-type MITE within the BrickBuilt element. The bottom panel depicts the CDS-to-CDs region BrickBuilt_11 from strain ATCC 33277, in which strain W83 has acquired a gene immediately upstream of the BrickBuilt element. Light grey boxing indicates completely identical sequence regions. Black lines or boxing indicates areas of aberrance (e.g. SNPs or additional IS-like element)

BrickBuilt_4 may best demonstrate the lingering ‘mobility’ of BrickBuilt elements within *P. gingivalis*. Strain HG66 shares the same locus with strain ATCC 33277. However, the TDC60 BrickBuilt_4 is not located between the same two genes (Fig. 7). No other mis-located BrickBuilt elements occur in strain TDC60 and the sequence of this element aligns closely with the ATCC 33277 and HG66 versions at this locus. Thus, it is probable that the BrickBuilt_4 homologue has been induced to transpose by or transposed with the surrounding ISPg1 elements. Additionally, no BrickBuilt_4 homologue is present in strain W83, adding to a mobility pattern of BrickBuilt_4 (Fig. 7). Importantly, BrickBuilt_4 is the only of these elements that has maintained perfect 12 bp TIR matches, increasing the likelihood that a surrogate transposase could act on the element. Further evidence of TSDs can be gleaned from this specific element by comparing the ‘filled’ and ‘empty’ sites between the strains. Strains ATCC 3377 and HG66, which contain the element at this locus, have a ‘GAAA’ tetranucleotide on each side of the intact TIRs. However, strains TDC60 and W83, which lack the element at this locus, only have a single ‘GAAA’ tetranucelotide copy.

With respect to *P. gulae* strains, BrickBuilt_5 demonstrates the possibility or history of mobility. At this site, 4 of the 11 *P. gulae* strains contain (and 7 lack) BrickBuilt copies. In the strains that contain an element a ‘AAAA’ TSD can be seen (‘AAAAA’ in *P. gingivalis* at that site). The *P. gulae* strains lacking an element at that site only have one ‘AAAA’ tetranucelotide. This site is completely conserved in the published *P. gingivalis* strains.

BrickBuilt_18 in the strains ATCC 33277 and HG66 contain/encompass MITE239_11 between nucleotides 659–904 of the sequence. Strain TDC60 has a gap where MITE239_11 occurs in the other two strains, while the flanking portions of the BrickBuilt match (Fig. 7). The strain W83 version at this site is diminutive, having been reduced to 1.5 copies of the 23 nt internal repeat. While strain W83 doesn’t harbor a MITE-within-a-MITE configuration at any locus, BrickBuilt_11 in strain W83 contains an ‘extra’ gene adjacent to the 5′ region of the element unlike any other strain (Fig. 7).

### Transcriptional expression of BrickBuilt

Repetitive and transposable elements are capable of modulating the genome stability and evolution of species [17, 19, 58, 66]. Interestingly, no endogenous plasmids have been found for *P. gingivalis* to date. The presence of many copies and types of repetitive and transposable elements could serve a quick way by which *P. gingivalis* could recombine/adapt to external stimuli beyond traditional host-directed transcriptional and translational controls [17, 18, 67–69].

#### Analysis of previously published data

**Fig. 8:**
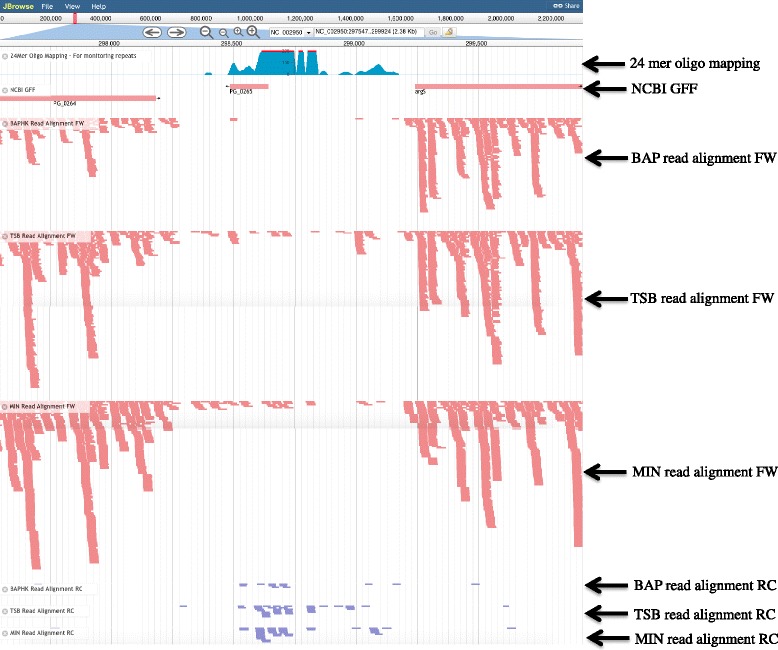
RNAseq display of transcripts of/from BrickBuilt_5 and surrounding area in strain W83 using JBrowse. Only uniquely mappable transcripts are displayed. Red horizontal lines correspond to forward strand-based transcripts from blood agar, tryptic soy and minimal media, respectively. Blue horizontal lines correspond to reverse strand-based transcripts from blood agar, tryptic soy and minimal media, respectively. (Full screen PDFs or screenshots are not currently possible with JBrowse, thus three different panels had to be compiled for the image. Direct link to data: http://bioinformatics.forsyth.org/jbrowse/index.html?data=PgRNAseq%2Fjson&loc=NC_002950%3A297655..299836&tracks=24Mer_Repeat%2Cncbigff%2Cbaphk_fw_bam%2Ctsb_fw_bam%2Cmin_fw_bam%2Cbaphk_rc_bam%2Ctsb_rc_bam%2Cmin_rc_bam&highlight=

Previous microarray and RNAseq studies have shown transcripts originating from within BrickBuilt elements, yet none characterized these regions in detail [9, 70]. Several of the microarray probes are themselves repetitive and many of the oligos/~20 mers used for identifying transcripts could map to multiple sites within the genome. Although BrickBuilt elements are highly conserved and repetitive, small variations due to SNPs, size of leader and tail regions, and the surrounding intergenic context make it possible to map at least some transcripts to the correct sites (Fig. 8 and Additional file 5: Figure S4). For situations where completely identical regions could produce the same transcript, the mapping programs and settings used will determine whether the transcripts are placed at one of the matching loci exclusively, distributed amongst the loci evenly, or left out of the results entirely. Importantly, the placement of any transcript at one or distributed across all of a given repetitive oligo/~20 mer sites suggests that at least one of the sites contributes active transcription.

Within the transcriptome transcript levels of individual BrickBuilt elements vary markedly and also vary according to growth medium, e.g. in/on minimal, tryptic soy, and blood media [9, 70]. Generally, transcripts from BrickBuilt regions are lowest on the blood-containing media. Transcript levels and distribution of transcripts of BrickBuilt elements, using strain W83 RNAseq data, can be grouped generally into three categories. Group one, displaying relatively high transcript levels throughout the element on only one strand bridging the entire CDS-to-CDS gap, includes BrickBuilt_1, 3, and 13. BrickBuilt_13, comprised only of the internal 23 nt repeats. The element’s expression correlates directly with that of the upstream gene, thus, the expression of BrickBuilt_13 could be completely due to transcriptional read-through from adjacent genes. Consistent with this, BrickBuilt_13-associated transcripts are all on the negative strand. Group two, displaying low to medium intermittent transcript in tryptic soy and minimal media but none on blood agar, includes BrickBuilt_2, 5, 6, 8, 10, 11, 12, 14, 15, 16, 17, and 19. Group three, displaying no transcript yet adjacent to upstream transcript that is well beyond an annotated CDS, includes only BrickBuilt_9.

Additional information about BrickBuilt elements and their surrounding regions can be garnered from the above microarrays and RNAseq studies as well as additional studies that have been carried out with *P. gingivalis* under defined conditions. High-density tiling microarray of *P. gingivalis* strain W83 by Chen *et al.* showed differential expression of BrickBuilt elements at several loci [9]. Using a W83 strain based microarray, genes PG0626 and PG0089 were found to be aberrant in strain ATCC 33277, corresponding to BrickBuilt_ 11 and BrickBuilt_ 19 loci. The area in and around BrickBuilt_10 was identified as a potential sRNA (sRNA35) by Philips et al. [71]. The highest expression of the putative sRNA35 occurred during mid-log cultures grown under hemin excess conditions after an initial period of hemin starvation. Under the experimental methods used by Philips et al., no other BrickBuilt loci were determined to be or be part of putative sRNAs expressed in response to hemin-variable growth conditions. BrickBuilt elements are not directly affected by FimR or LuxS regulation [72, 73]. However, genes surrounding BrickBuilt elements are regulated by LuxS. Lack of expression as well as partial expression of annotated genes surrounding BrickBuilt elements is evident from *P. gingivalis* strain W83 transcriptomic analyses by Hovik et al. [70]. Five of the conserved 30 genes flanking BrickBuilt elements in the W83 genome are predicted to not be expressed and 11 (including 3 of the 5 ‘not expressed’) give partial or abortive transcripts in blood, tryptic soy or minimal media.

The genomic association with haem biosynthesis and pigmentation-associated genes in conjunction with transcriptional data from RNAseq and microarray studies may point to regulation of BrickBuilt regions by haem or iron. DNA tandem repeats have been shown previously to affect transcription of iron and haem-associated genes [18, 74].

#### E. coli expression vectors

**Fig. 9:**
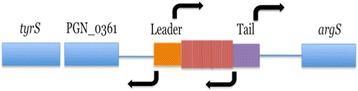
Model of promoter capabilities of BrickBuilt_5 based on *lacZ* promoter probe ability. Bi-directional promoters are present in both the leader and tail segments of BrickBuilt_5. As such, at this locus antisense transcripts may be produced toward PGN_0361, sense transcripts produced toward arginyl-tRNA synthetase (*argS*), and transcripts of the 23 nucleotide repeat regions within the element may be produced from both strands. The distance from the tail to *argS* is less than 100 nt and may be or contain the promoter for *argS*

**Fig. 10:**
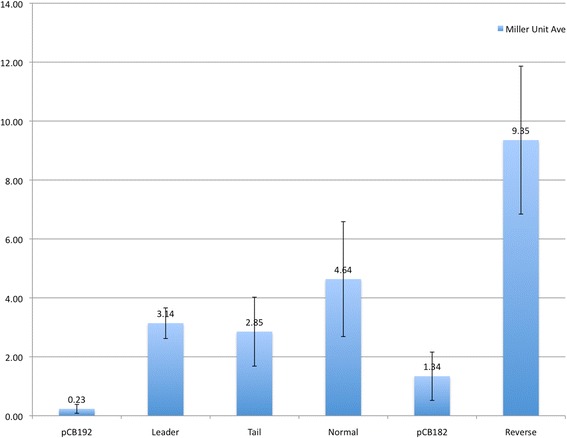
ONPG assays for promoter capabilities of BrickBuilt_5 based on *lacZ* promoter probe constructs. Promoter-less *lacZ* backbone vectors pCB182 and pCB192 give low apparent β –Galactosidase activity. β –Galactosidase activity measured through ONPG cleavage after 3 h incubation at 28 °C. Error bars represent standard deviation between biological replicates in triplicate. Statistical significance determined by *t*-test (*p* < 0.05)

Promoter probe vectors pCB182 and pCB192 were used to determine the potential for transcription and transcriptional regulation of the full BrickBuilt element and segments. Four potential promoter sites were hypothesized based on previous RNAseq and microarray data (Fig. 9). Four configurations of the leader, tail and element were constructed using BrickBuilt_5 as a template: full element in leader-to-tail orientation (‘normal’, with tail abutting *lacZ*); full element in tail-to-leader orientation (‘reverse’, with flipped leader abutting *lacZ*); tail-only in reverse orientation; and leader-only in forward orientation. The reverse orientation of the full element, with the beginning of the leader abutting the promoter-less *lacZ*, displayed the greatest promoter activity of the four constructs (Fig. 10). All four constructs displayed statistically significant expression under heterologous expression in *E. coli*. No expression from the vectors lacking inserts was seen on plates with X-gal, while each insert-containing construct showed blue colonies due to expression by 24 h (Additional file 6: Figure S5). Expression from these constructs demonstrates bi-directional promoter ability (when tested in an *E. coli* system) within the tail segment as well as in the leader segment facing out of the element.

## Conclusions

We identified and provided preliminary characterization of a genetic element, ‘BrickBuilt’, in the genome of *Porphyromonas gingivalis*. BrickBuilt appears to be a MITE-like element that has trapped a 23 nt direct repeat; propagating itself and the direct repeat throughout the genome. From promoter-less *lacZ* assays and analyses of previous microarray and RNAseq data we determined certain BrickBuilt elements contain promoter elements capable of bi-directional transcription. Given the element’s exclusively intergenic locations and surrounding gene directionality, these transcripts may serve to regulate expression of surrounding genes. Relative stability of locations, overall copy number and expression levels of the elements throughout the sequenced *P. gingivalis* genomes point to neutral or advantageous maintenance of BrickBuilt.

Further sequencing projects and phylogenomics will be necessary to determine which other species and strains contain the BrickBuilt element and at what evolutionary point these species and/or strains diverged. Additionally, strain-specific experimental evolution and plasmid-based recombination systems could be employed to determine mutation rates within BrickBuilt in comparison to the rest of the genome, whether and how 23 nt repeats expand and contract at a given locus, and how recombinogenic the elements are.

With respect to ‘mobility’ of a whole or partial BrickBuilt element, several experimental setups could be considered. First, inducing expression of endogenous transposases in order to mobilize BrickBuilt elements. One would need to initially determine under what conditions each transposase type in *P. gingivalis* is expressed, then induce expression and either PCR or sequence target BrickBuilt locations. Additionally, whole genome sequence could be employed to find the locations of element movement or duplication. Second, exogenous transposases could be expressed in *P. gingivalis*. Given the specificity of some transposases, a panel may need to be tested. Third, BrickBuilt elements could be introduced on plasmids into other bacterial species, specifically other *Bacteroidetes*, in order to try to obtain insertion into the heterologous host.

Adding BrickBuilt to the list of transposable and repetitive elements types in *P. gingivalis* brings the current total to 4 MITEs (or MITE-like elements), 11 ISs, 2 Ctn and 1 Tn. The ORFs and total base pairs encompassed by these elements constitute an impressive proportion of the genome. When compiled, the total percent of the *P. gingivalis* genome encoded by MITEs is 1 %; 0.44 % from BrickBuilt elements, 0.39 % from MITEPgRS elements, and the remaining 0.17 % from MITE700 and MITE239 family elements.

The ability of several of these elements being involved in genome evolution has been established [47, 48]. However, the full effects of these elements on genome stability and evolution as well as transcriptional, translational and post-translational response to stimuli remains to be experimentally determined.

## Methods

### Genomes/Strains

Genome sequence FASTA and GenBank files were downloaded from the NCBI database. At the time of this research, strains ATCC 33277, TDC60, W83, HG66 and JCVI SC001 were available as completed sequencing and assembly projects (ATCC 33277, TDC60 and W83 as ‘gapless chromosome’ status, HG66 as a single contig, and JCVI SC001 as a draft of many stitched contigs) [10, 13–16]. The five sequenced wild-type strains are disparate based on origin or lineage: W83 isolated in Germany (1950’s) from an oral lesion; ATCC 33277 was isolated in the USA (1980’s) from subgingival plaque; TDC60 was isolated in Japan (2011) from an oral lesion; HG66 isolated in the USA (1989) from a dental school patient; and JCVI SC001 was isolated in the USA (2013) from a hospital sink. The sequencing projects utilized different sequencing and assembly methods; each providing a *de novo* assembly. The JCVI SC001 genome sequence contained unidentified bases and residual gaps in the sequencing after the completed project.

### Sequence analysis, clustering, alignment, phylogeneics/phylogenomics

NCBI BLAST suites were utilized to determine locations, structure and potential protein-coding capacity of the MITEs [43]. Query inputs were FASTA sequences taken directly from NCBI genome sequencing projects. For initial characterizations prior to determining species-specificity of the elements, the entire NCBI sequence database was queried. Following determination that the elements were only found (as of 11/2013) in the genomes of *P. gingivalis* strains, subsequence queries were focused to either the *P. gingivalis* species as a whole or specific *P. gingivalis* strains. Megablast, discontiguous megablast and BLASTn program selections for search optimization were all used in determining species-specificity as well as genome localizations.

MultAlin, Clustal Omega and the MEME suite were used to perform DNA-based and amino acid-based multiple alignments of the MITEs to determine conserved nucleotides and the start and stop points of the elements as well as proteins surrounding the MITEs. Amino acid-based alignments were used to determine whether the surrounding genes had structural domains at either the 5′ or 3′ ends that could potentially account for or facilitate MITE localization [75–77].

The BioCyc sequence pattern search tool ‘PatMatch’ was used to determine the number and genomic location of *P. gingivalis* MITEs in strain ATCC 33277 [78]. PatMatch indentifies potential sites given variations in the consensus sequences of the MITE direct repeats, TIRs, ‘leader’ and ‘tail’ regions because different mismatch numbers are allowed. Query inputs were nucleotide consensus sequences determined for each of the given parts of the MITE. Both DNA strands, as well as intergenic and coding sequences, were queried separately. Mismatches of ‘0’ through ‘3’ were allowed, with the constraint of the ‘mismatch type’ being a substitution.

The Tandem Repeats Database software was used for determining all types of tandem repeat elements in the *P. gingivalis* genomes (strains ATCC 33277 and W83 hosted on the server as of 12/2013), and to determine whether the tandem repeats or MITE as a whole was conserved in other sequenced species [79]. BLAST query of the entire bacterial and viral tandem repeat database was carried out using the FASTA sequence downloaded from NCBI for *P. gingivalis* strain ATCC 33277 MITE. The Tandem Repeats Finder software was used to determine the composition of the *P. gingivalis* tandem repeat element [80]. ‘Basic’ sequence analysis was selected for queries. Tandem Repeats Finder was also used to determine repeat conservation within and between loci as well as where a given element started and ended.

The Geneious software platform (version R8) was used to download, store, deposit, manipulate and query *P. gingivalis* genomes and BrickBuilt MITEs [81].

### MITE and surrounding coding Sequences’ nucleic acid and protein motif analysis

The Pfam and InterProScan databases and programs software were used to determine the presence and characteristics of nucleic acid and protein motifs [82, 83]. Query inputs were FASTA sequences from NCBI download files. For Pfam, an E-value of 1.0 and checking Pfam-B motifs were selected options prior to submission.

ExPASy Translate Tool software was used to determine whether the MITEs potentially encoded proteins, and thus are not strictly nucleic acid elements [84]. Genetic code option ‘standard’ was used for all queries. All six possible frames of translation were considered.

Modeling and structure prediction programs Mfold, RNAstructure and RegRNA2.0 were used to predict potential 2-D structures formed by MITE DNA and RNA [53–55]. Default options for programs in regard to structure prediction were chosen.

### Cloning and reporter strains, media and growth conditions

*Escherichia coli* DH5α and TOP10 were used for cloning, plasmid maintenance and transcriptional assays. Ampicillin (100 μg/ml) was used when appropriate for prevention of contamination as well as isolation and maintenance of transformants containing plasmids. Strains were grown and maintained on LB [Lennox] agar or in LB [Lennox] broth (Invitrogen).

PCR primers containing BamHI and XmaI (NEB) restriction sites were designed immediately flanking the BrickBuilt_5 MITE associated with PGN_0361 (Additional file 2: Table S2). PCR products were generated using GoTaqLong Master Mix (Promega) and resultant bands were cloned into vector pCR TOPO-XL (Invitrogen), which was transformed into *E. coli* DH5α. Transformants were selected for kanamycin resistance and clones were confirmed by restriction digest and sequencing.

To generate constructs using the promoter probe vectors pCB182 and pCB192, pCR2.1 and pCR-TOPO-XL cloned BrickBuilt MITE constructs and pCB182/pCB192 were double-digested with BamHI (NEB) and XbaI (NEB) or BamHI and XmaI (NEB) and then transformed into *E. coli* TOP10. Transformants were selected for ampicillin resistance generated by an insertion event. Clones were confirmed by restriction digest and sequencing.

### Transcriptional analyses

BROP, specifically the ‘Genomics Tools for Oral Pathogens’ and ‘Microbial Transcriptome Database’ sections of the resource, were used to determine genome location, characteristics of coding sequencings surrounding MITEs, differences between strains as well as transcriptome data [85]. Over the course of the research, two different variations of the RNAseq data for *P. gingivalis* strain W83 were supported, one directly on BROP and then a later form on JBrowse. The JBrowse form gives greater functionality in displaying data and visualization [86]. Under the ‘Genomics Tools for Oral Pathogens’ subset, the ‘GenomeViewer’ function was used to compare genome arrangements of *P. gingivalis* strains ATCC 33277 and W83 with relation to MITEs, as well as display the previously performed microarray data (under the strain W83 section) for MITE-associated genome areas under the three different nutrient conditions (the same conditions performed in the RNAseq) [9, 70].

#### β –galactosidase assays

*Escherichia coli* strains were grown and maintained in Luria-Bertani (LB) media supplemented with ampicillin (100 μg/l) as required. PCR primers and synthesized oligos used for strain constructions are listed in Additional file 2: Table S2. The pCB182 and pCB192 vectors lack promoters but contain translational start codons [87]. As such, gene expression of *lacZ*, and in turn protein expression of LacZ read out through β –galactosidase activity, should be the result of promoter activity from fragments cloned into the vector. β -galactosidase assays were performed under plate-based (X-Gal) and broth-based (ONPG) setups. For plate-based assays, frozen stock cultures of the BrickBuilt MITE derivatives transcriptionally fused to *lacZ* in their respective *E. coli* strains were plated onto LB agar containing X-gal and ampicillin. For broth-based assays, cultures of the BrickBuilt MITE derivatives transcriptionally fused to *lacZ* in their respective *E. coli* strains were grown in LB broth for 3 h with shaking at 37 °C. An aliquot of each culture (500 μl) was added to a lysis and assay solution mixture (500 μl), vortexed, and then incubated at 28 °C for 3 h. Color development was measured spectrophotometrically at OD_420_ nm and cell debris at OD_550_ nm. Respective Miller units were calculated as previously described [88].

All data, genome sequences as well as RNAseq and microarray, are currently available in public repositories and publications related to these data have been referenced. Locations of the MITE sequences in *P. gingivalis* and *P. gulae* strains will be deposited to NCBI such that identifiers and notes can be amended to the graphic outputs of sequence files.

## Additional files

Additional file 1: Figure S1. Tandem Repeat Finder analysis of *P. gingivalis* strain ATCC 33277 BrickBuilt_5. Overall statistics of the repeats/repeat region found within BrickBuilt_5; 23 nt repeat indicies of the element relative to the entire element, period size, copy number, consensus size, percent matches, percent InDels, alignment score, percent composition for each nucleotide and entropy measure based on percent composition. The individual locations of mismatches and InDels within BrickBuilt_5 are shown as positions marked by stars (*). (PDF 92 kb) Additional file 2: Table S1. Loci and nucleotide sites of BrickBuilt elements across four sequenced and annotated *P. gingivalis* strains. BrickBuilt elements are situated intergenically between the genes noted. Grayed-out boxes represent loci at which BrickBuilt is aberrant. **Table S2.** Primers for sequencing and cloning. Sequence of oligos ordered for cloning directly into promoter-probe vectors. (XLSX 66 kb) Additional file 3: Figure S2.
*P. gingivalis* strain SJD2 assembly showing an ‘assembly gap’ at the site of BrickBuilt elements from strains ATCC 33277, W83, TDC60 and HG66. The same genes flanking the assembly gap are found flanking BrickBuilt_11 in strains ATCC 33277, W83, TDC60 and HG66. The top dark green track depicts individual contigs (a total of 140 for this strain), the second dark green track depicts individual scaffolds (a total of 117 for this strain), the yellow track shows predicted coding sequences, the light green track shows predicted genes, and the bottom track shows the assembly gap as a rightward-pointing triangle. (PNG 19 kb) Additional file 4: Figure S3. BrickBuilt_5 region MAFFT alignment and PHYML tree of *P. gulae* and *P. gingivalis* strains. All COT_052 *P. gulae* strains were sequenced/deposited during the preparation of the manuscript. Additionally, all *P. gulae* strains are currently scaffold or contig assemblies; none are completed chromosomes and thus are also not available for default BLASTn query on NCBI. The aligned bases between 2,500-3,800 contain the BrickBuilt_5 MITE; flanking regions contain the same 2 upstream and downstream genes in all strains. Of the 12 nodes in the tree, 8 have a bootstrap value of greater than 85 (100 bootstrap iterations). In the ‘consensus identity’ track, green indicates sites of complete conservation, yellow of partial conservation and red of little conservation. Within each of the 15 strain tracks the black lines or blocks indicate sites that deviate from the consensus at that given site. (PDF 108 kb) Additional file 5: Figure S4. Microarray display of transcripts in BROP MTD database of BrickBuilt_5 and surrounding area in strain W83. Tracts represent positive and negative strand blood agar (top), tryptic soy broth (middle) and minimal media (bottom), respectively. BrickBuilt_5 noted with black bracket. Additional file 6: Figure S5. X-gal and ONPG assays of promoter capabilities of BrickBuilt_5 based on *lacZ* promoter probe constructs. Promoter-less *lacZ* vectors pCB182 and pCB192 give no apparent β –Galactosidase activity. BrickBuilt_5 leader and tail oligos (Eurofins Operon) were cloned into vector pCB192. Full-length BrickBuilt_5 was cloned into pCB192, ‘Normal’, with the tail segment of the element upstream of *lacZ* facing in the same orientation (tail abutting *lacZ*). Full-length BrickBuilt_5 was cloned into pCB182, ‘Reverse’, with the leader segment of the element upstream of *lacZ* facing in the same orientation (flipped leader abutting *lacZ*). X-gal activity visualized after 24 h incubation. Top of two liquid assays of ONPG activity visualized after 3 h incubation; bottom after 24 h incubation.

## Abbreviations

MITE: Miniature Inverted-repeat Transposable Element
RE: Repetitive Element
TE: Transposable Element
TIR: Terminal Inverted Repeat
TSD: Target Site Duplication
PCR: Polymerase Chain Reaction
BROP: Bioinformatics Resource Oral Pathogens
MTD: Microbial Transcriptome Database
BLAST: Basic Local Alignment Search Tool
OD: Optical Density
X-gal: 5-bromo-4-chloro-3-indolyl-beta-D-galacto-pyranoside
ONPG: O-Nitrophenyl-β-D-galactopyranoside
