# Supplementary material for: Identification and characterization of a minisatellite contained within a novel miniature inverted-repeat transposable element (MITE) of Porphyromonas gingivalis
Source: Mob DNA. 2015 Oct 6;6:18. doi: 10.1186/s13100-015-0049-1 (PMC4596501; doi:10.1186/s13100-015-0049-1)
Supplement: Additional file 1: Figure S1. — Tandem Repeat Finder analysis of P. gingivalis strain ATCC 33277 BrickBuilt_5. Overall statistics of the repeats/repeat region found within BrickBuilt_5; 23 nt repeat indicies of the element relative to the entire element, period size, copy number, consensus size, percent matches, percent InDels, alignment score, percent composition for each nucleotide and entropy measure based on percent composition. The individual locations of mismatches and InDels within BrickBuilt_5 are shown as positions marked by stars (*). (PDF 92 kb) [file 13100_2015_49_MOESM1_ESM.pdf]

Indices	Period Size	Copy Number	Consensus Size	Percent Matches	Percent Indels	Score	A	C	G	T	Entropy (0-2)
250--683	23	18.9	23	95	0	724	27	25	15	31	1.96

```
                        *
250 AGACCATAGTATCCTCTCATGTG
  1 AGACCATAGTATCCTCTCATATG

                      *
273 AGACCATAGTATCCTCTCGTATG
  1 AGACCATAGTATCCTCTCATATG

                        *
296 AGACCATAGTATCCTCTCATGTG
  1 AGACCATAGTATCCTCTCATATG

       *                *
319 AGATCATAGTATCCTCTCATGTG
  1 AGACCATAGTATCCTCTCATATG

       *              * *
342 AGATCATAGTATCCTCTCTTGTG
  1 AGACCATAGTATCCTCTCATATG

                        *
365 AGACCATAGTATCCTCTCATGTG
  1 AGACCATAGTATCCTCTCATATG

                        *
388 AGACCATAGTATCCTCTCATGTG
  1 AGACCATAGTATCCTCTCATATG

                      *
411 AGACCATAGTATCCTCTCGTATG
  1 AGACCATAGTATCCTCTCATATG

434 AGACCATAGTATCCTCTCATATG
  1 AGACCATAGTATCCTCTCATATG

                        *
457 AGACCATAGTATCCTCTCATGTG
  1 AGACCATAGTATCCTCTCATATG

480 AGACCATAGTATCCTCTCATATG
  1 AGACCATAGTATCCTCTCATATG

503 AGACCATAGTATCCTCTCATATG
  1 AGACCATAGTATCCTCTCATATG

526 AGACCATAGTATCCTCTCATATG
  1 AGACCATAGTATCCTCTCATATG

                      *
549 AGACCATAGTATCCTCTCGTATG
  1 AGACCATAGTATCCTCTCATATG

572 AGACCATAGTATCCTCTCATATG
  1 AGACCATAGTATCCTCTCATATG

            *
595 AGACCATAATATCCTCTCATATG
  1 AGACCATAGTATCCTCTCATATG

                      *
618 AGACCATAGTATCCTCTCGTATG
  1 AGACCATAGTATCCTCTCATATG

                      *
641 AGACCATAGTATCCTCTCGTATG
  1 AGACCATAGTATCCTCTCATATG

664 AGACCATAGTATCCTCTCAT
  1 AGACCATAGTATCCTCTCAT
```
