# Supplementary figures and images for: Identification and characterization of a minisatellite contained within a novel miniature inverted-repeat transposable element (MITE) of Porphyromonas gingivalis
Source: Mob DNA. 2015 Oct 6;6:18. doi: 10.1186/s13100-015-0049-1 (PMC4596501; doi:10.1186/s13100-015-0049-1)

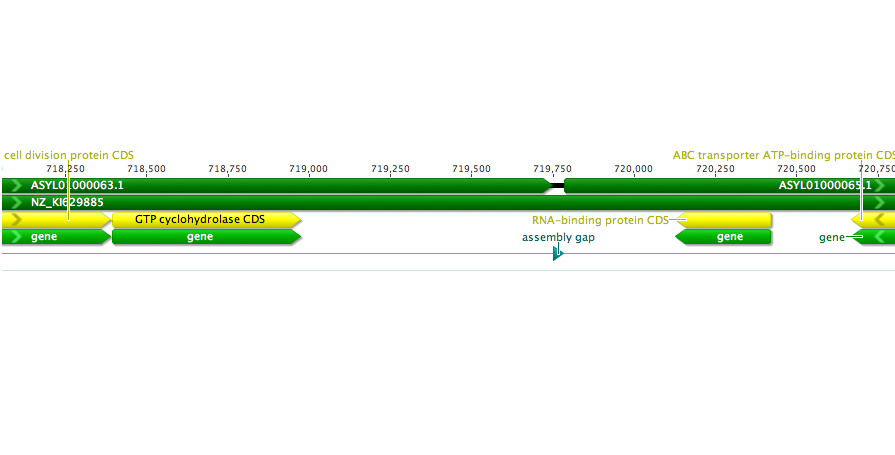

Supplement: Additional file 3: Figure S2. — P. gingivalis strain SJD2 assembly showing an ‘assembly gap’ at the site of BrickBuilt elements from strains ATCC 33277, W83, TDC60 and HG66. The same genes flanking the assembly gap are found flanking BrickBuilt_11 in strains ATCC 33277, W83, TDC60 and HG66. The top dark green track depicts individual contigs (a total of 140 for this strain), the second dark green track depicts individual scaffolds (a total of 117 for this strain), the yellow track shows predicted coding sequences, the light green track shows predicted genes, and the bottom track shows the assembly gap as a rightward-pointing triangle. (PNG 19 kb) [file 13100_2015_49_MOESM3_ESM.png]

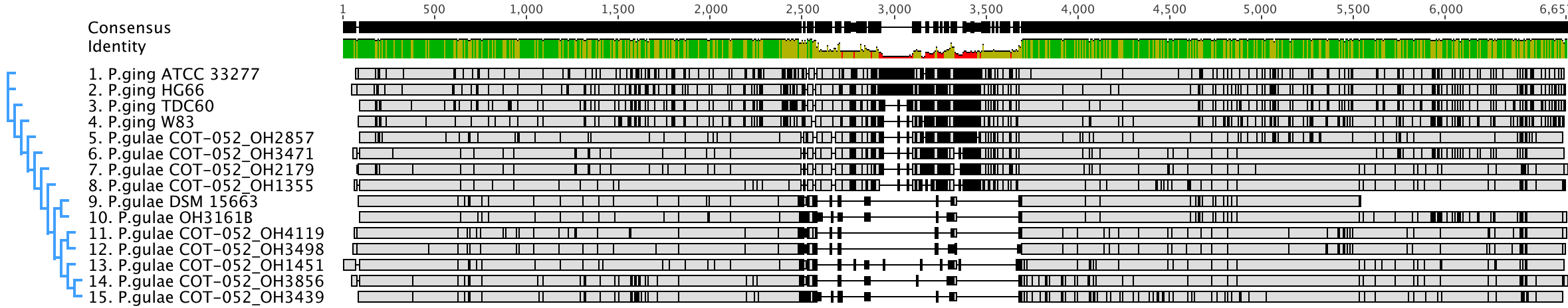

Supplement: Additional file 4: Figure S3. — BrickBuilt_5 region MAFFT alignment and PHYML tree of P. gulae and P. gingivalis strains. All COT_052 P. gulae strains were sequenced/deposited during the preparation of the manuscript. Additionally, all P. gulae strains are currently scaffold or contig assemblies; none are completed chromosomes and thus are also not available for default BLASTn query on NCBI. The aligned bases between 2,500-3,800 contain the BrickBuilt_5 MITE; flanking regions contain the same 2 upstream and downstream genes in all strains. Of the 12 nodes in the tree, 8 have a bootstrap value of greater than 85 (100 bootstrap iterations). In the ‘consensus identity’ track, green indicates sites of complete conservation, yellow of partial conservation and red of little conservation. Within each of the 15 strain tracks the black lines or blocks indicate sites that deviate from the consensus at that given site. (PDF 108 kb) [file 13100_2015_49_MOESM4_ESM.pdf]

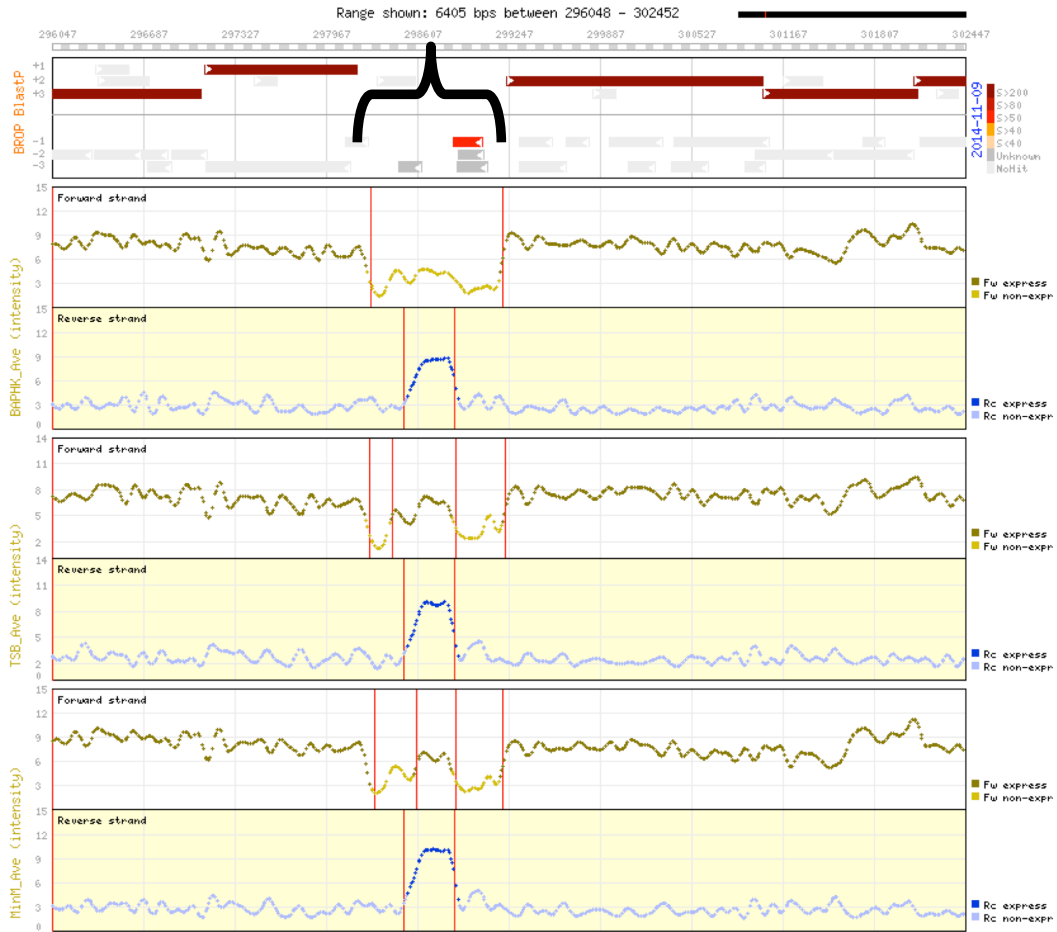

Supplement: Additional file 5: Figure S4. — Microarray display of transcripts in BROP MTD database of BrickBuilt_5 and surrounding area in strain W83. Tracts represent positive and negative strand blood agar (top), tryptic soy broth (middle) and minimal media (bottom), respectively. BrickBuilt_5 noted with black bracket. [file 13100_2015_49_MOESM5_ESM.pdf]

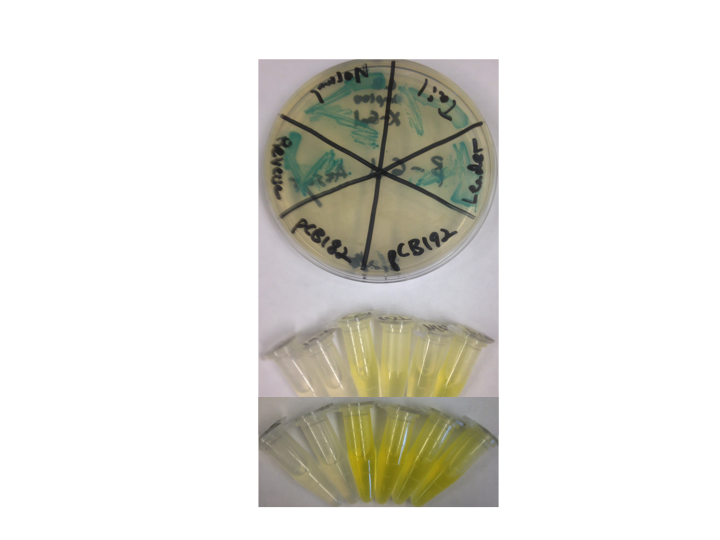

Supplement: Additional file 6: Figure S5. — X-gal and ONPG assays of promoter capabilities of BrickBuilt_5 based on lacZ promoter probe constructs. Promoter-less lacZ vectors pCB182 and pCB192 give no apparent β –Galactosidase activity. BrickBuilt_5 leader and tail oligos (Eurofins Operon) were cloned into vector pCB192. Full-length BrickBuilt_5 was cloned into pCB192, ‘Normal’, with the tail segment of the element upstream of lacZ facing in the same orientation (tail abutting lacZ). Full-length BrickBuilt_5 was cloned into pCB182, ‘Reverse’, with the leader segment of the element upstream of lacZ facing in the same orientation (flipped leader abutting lacZ). X-gal activity visualized after 24 h incubation. Top of two liquid assays of ONPG activity visualized after 3 h incubation; bottom after 24 h incubation. [file 13100_2015_49_MOESM6_ESM.png]
